# Prognostic and predictive value of ALDH1, SOX2 and SSEA-4 in bladder cancer

**DOI:** 10.1038/s41598-021-93245-1

**Published:** 2021-07-01

**Authors:** Matias Blomqvist, Ilmari Koskinen, Eliisa Löyttyniemi, Tuomas Mirtti, Peter J. Boström, Pekka Taimen

**Affiliations:** 1 Institute of Biomedicine and FICAN West Cancer Centre, University of Turku, Turku, Finland; 2 Department of Urology, Helsinki University Hospital and University of Helsinki, Helsinki, Finland; 3 Department of Biostatistics, University of Turku, Turku, Finland; 4 Department of Pathology, University of Helsinki and Helsinki University Hospital, Helsinki, Finland; 5 Department of Urology and FICAN West Cancer Centre, University of Turku and Turku University Hospital, Turku, Finland; 6 Department of Pathology, Turku University Hospital, Turku, Finland

**Keywords:** Bladder cancer, Cancer stem cells, Tumour biomarkers

## Abstract

Transurethral resection of bladder tumor (TUR-BT) and radical cystectomy (RC) are standard treatment options for bladder cancer (BC). Neoadjuvant chemotherapy (NAC) prior to RC improves outcome of some patients but currently there are no valid biomarkers to identify patients who benefit from NAC. Presence of cancer stem cells (CSC) has been associated with poor outcome and resistance to chemotherapy in various cancers. Here we studied the expression of stem cell markers ALDH1, SOX2 and SSEA-4 with immunohistochemistry in tissue microarray material consisting of 195 BC patients treated with RC and 74 patients treated with TUR-BT followed by NAC and RC. Post-operative follow-up data of up to 22 years was used. Negative to weak cytoplasmic SOX2 staining was associated with lymphovascular invasion and non-organ confined disease. It was also associated with shortened cancer-specific survival, but the finding was not statistically significant. Contrary to previous reports, none of the other tested biomarkers were associated with cancer-specific mortality or clinicopathological characteristics. Neither were they associated with response to NAC. Despite the promising results of previously published studies, our results suggest that CSC markers ALDH1, SOX2 and SSEA-4 have little if any prognostic or predictive value in BC treated with RC.

## Introduction

Bladder cancer (BC) is the 10th most common cancer worldwide with estimated 549,000 new cases and 200,000 deaths annually^1^. The most important risk factors for BC include tobacco use, aging, male gender, and exposure to certain chemicals^2^.

Non-muscle invasive bladder cancer (NMIBC, stage pTa-T1 tumors) is routinely treated with transurethral resection of bladder tumor (TUR-BT) and various intravesical treatments. However, up to 78% of these tumors recur^3^. For muscle-invasive bladder cancer (MIBC, stage T2 or higher) radical cystectomy (RC) with pelvic lymphadenectomy and cisplatin-based neoadjuvant chemotherapy (NAC) are preferred when applicable. NAC improves 5-year progression free and overall survival in locally invasive disease by 8%^2^, but to date there are no immunohistochemical or other biomarkers to distinguish aggressive tumors from non-aggressive tumors, or chemotherapy responders from non-responders. Therefore, there is an unmet need of novel tools for patient stratification and selection.

Cancer stem cell theory dictates that similar to normal tissues, cancer tissue has a subpopulation of cancer stem cells (CSCs) or tumor-initiating cells that drive the proliferation of the tumor and initiate metastasis^4–7^. Several molecular biomarkers have been used to identify CSCs. However, many gene products are associated with CSC-like phenotype and none of them is universal for all cancer types. Sox2, a family member of sex determining region Y (SRY) -box genes, is a key regulator of pluripotency in stem cells^8–10^ and has been linked to poor survival in various malignancies including BC^11–14^. ALDH1, a member of aldehyde dehydrogenase subfamily, regulates pluripotency via retinoic acid pathway and has been implicated as a CSC biomarker^15,16^. ALDH1 has also been associated with poor outcome and clinicopathological features in BC^17–19^. Stage-specific embryonic antigen 4 (SSEA-4) is a ganglioside present on the cell surface of embryonic stem cells and synthesized by the enzyme ST3Gal II^20,21^. Increased expression of SSEA-4 has been linked to poor survival in various cancers including lung, breast, prostate and brain^22–25^ and SSEA-4 is also considered a potential drug target in glioblastoma multiforme^23^. BC cell line HTB-9 (5637) has been reported to express SSEA-4^26,27^ but so far, the expression of SSEA-4 has not been studied in clinical BC specimens in more detail.

Importantly, there is data that the number of CSCs is enriched in cisplatin-resistant subclone of T24 bladder cancer cell line (DR-24T) compared to parental cells and these cells were more tumorigenic in mouse xenograft studies^28^. Prompted by aforementioned findings, we studied the expression of three CSC-related biomarkers, ALDH1, SOX2 and SSEA-4 in two different cohorts of BC patients treated with either RC or TUR-BT, followed by NAC and RC. To our surprise, none of tested markers were statistically significantly associated with disease progression in our patient cohorts. Neither were there significant correlations with patients’ NAC response implying that ALDH1, SOX2 and SSEA-4 play no major role in the progression of BC and/or multiple other factors determine the aggressiveness of the disease.

## Results

### Patient cohort

Patient characteristics of RC only cohort (n = 195) and NAC cohort (n = 74) are shown in Table 1. Patients included into NAC cohort underwent TUR-BT and received either 2–6 cycles of cisplatin-gemcitabine (68 of 74 patients) or carboplatin-gemcitabine (6 of 74 patients) prior to cystectomy.

**Table 1:** Baseline characteristics of patients.

	RC only cohort, n (%)	NAC cohort, n (%)
Patients total	195 (100)	74 (100)
**Age**		
Mean/median (range)	63/65 (35–80)	64/65 (47–76)
**Gender**		
Male	160 (82)	62 (84)
**Smoking**		
Yes	99 (51)	55 (74)
**pT category in cystectomy**		
T0	0 (0)	29 (39)
pTa, pTis, pT1	94 (48)	16 (22)
pT2	41 (21)	13 (18)
pT3	48 (25)	11 (15)
pT4	12 (6)	5 (7)
**Lymphovascular invasion in RC specimen**		
Yes	66 (34)	11 (15)
**Grade (WHO 1973)**^**a**^		
G1	9 (5)	1 (1)
G2	69 (35)	18 (24)
G3	115 (59)	50 (68)
Data not available/applicable	2 (1)	5 (7)
**Grade (WHO/ISUP 2004) **^**a**^		
Low grade	9 (5)	1 (1)
High grade	184 (94)	68 (92)
Data not available/applicable	2 (1)	5 (7)
**Histological type**^**a**^		
Urothelial	187 (96)	69 (93)
Squamous Cell Carcinoma	5 (3)	1 (1)
Adenocarcinoma	3 (2)	0 (0)
Small cell/neuroendocrine	0 (0)	4 (7)
**Adjuvant chemotherapy**		
Yes	1 (0)	7 (9)
**Follow-up time (years)**		
Mean/median (range)	5.5/3.7 (0.1–22)	3.2/3.3 (0.3–8.3)
**Status**		
Alive, no evidence of disease	50 (26)	55 (74)
Alive with recurrence	1 (0)	2 (3)
Death from bladder cancer	70 (36)	14 (19)
Death from other reason	55 (28)	3 (4)
Lost for follow-up	19 (10)	0 (0)
**Pathological response to NAC**		
Complete response (pT0)	N/A	28 (38)
Partial response (pT1/pTa/pTis)	N/A	15 (20)
No response	N/A	11 (15)
Progression (pT3 and/or N+)	N/A	19 (26)
Data not available	N/A	1 (1)

^a^Based on histopathological review of cystectomy specimen for RC cohort and TUR-BT for NAC cohort, respectively.

### Staining patterns

**Figure 1:**
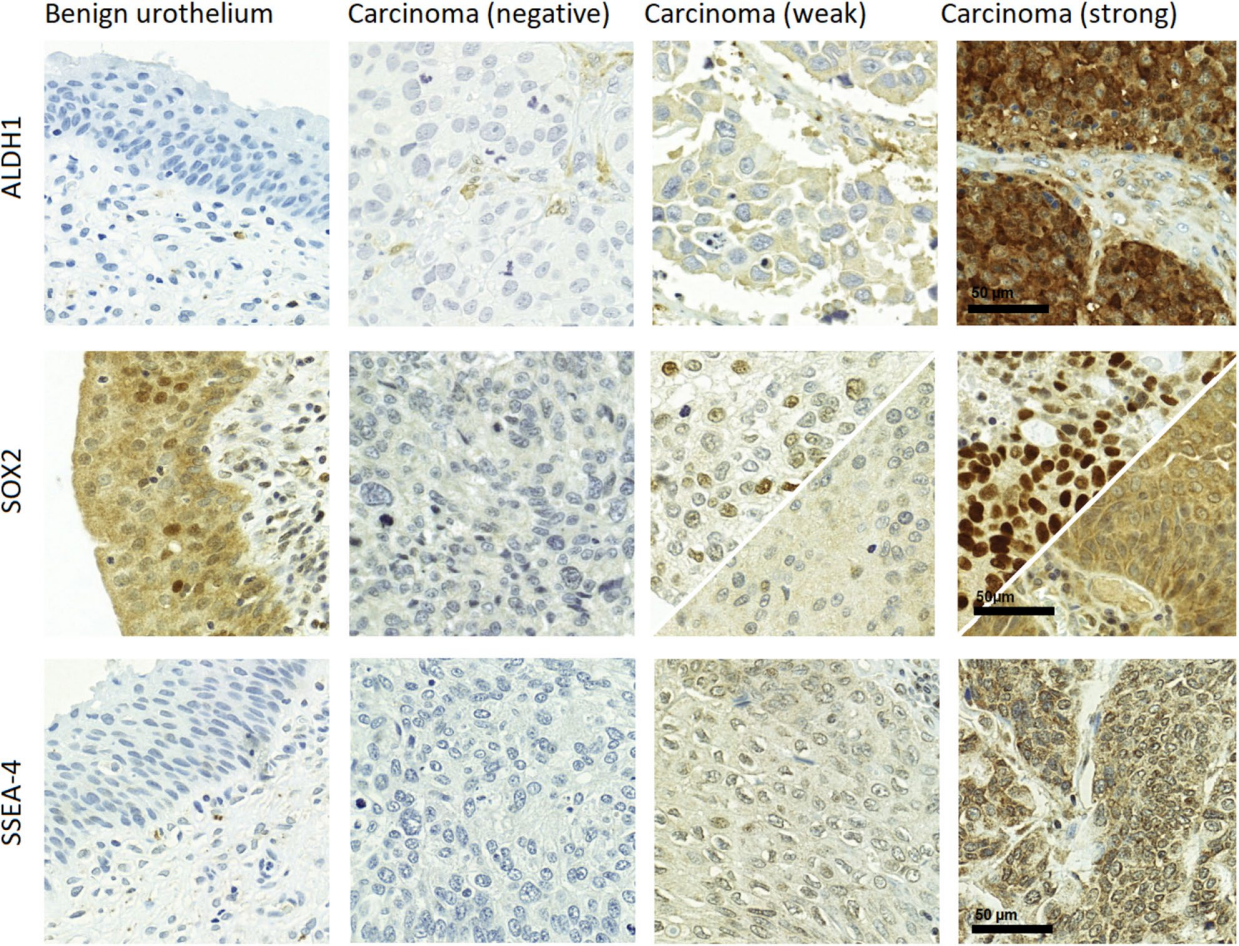
Representative examples of normal benign urothelium and negatively, weakly and strongly stained carcinoma tissue for ALDH1, SOX2 and SSEA-4. For SOX2, both weak/strong cytoplasmic and nuclear expression patterns are shown (nuclear staining pattern on the top half and cytoplasmic on the bottom).

Immunohistochemical ALDH1, SOX2 and SSEA-4 stainings were successful from 173, 167, and 170 patients included into RC cohort, respectively. Representative examples of negative, weakly, and strongly stained carcinoma cells, as well as typical staining pattern on benign urothelium are shown in Fig. 1. None of the markers were associated with age, gender or smoking.

For ALDH1, there were clear differences in cytoplasmic staining intensity between individual patients’ tumors (from negative to strongly expressing cells) while the normal urothelium was generally negative (Fig. 1). H-score of ALDH1 was greater than 0 in 48 (28%) tumors and 4 or more in 22 (13%) tumors (Table 2).

**Table 2:** Results of immunohistochemical staining from the RC-only cohort.

	Proportion of positive cells, n (%)					Staining intensity, n (%)			H-score, n (%)^a^			
Marker	0	1–25	26–50	51–75	76–100	Neg.^b^	Weak	Strong	0	1–3	4–6	7–8
ALDH1	125 (72)	25 (14)	9 (5)	4 (2)	10 (6)	125 (72)	12 (7)	36 (21)	125 (72)	26 (15)	12 (7)	10 (6)
SOX2 (N^c^)	48 (29)	60 (36)	16 (10)	15 (9)	28 (11)	48 (29)	16 (10)	103 (62)	48 (29)	60 (36)	31 (19)	28 (17)
SOX2 (C^d^)	26 (16)	13 (8)	11 (7)	23 (14)	94 (56)	26 (16)	26 (16)	115 (69)	26 (16)	27 (16)	24 (14)	90 (54)
SSEA-4	105 (62)	16 (9)	8 (5)	14 (8)	27 (16)	105 (62)	37 (22)	28 (16)	105 (62)	31 (18)	18 (11)	15 (9)

^a^H-score (0–8) = staining intensity score (0–2) multiplied by proportion score (0–4).

^b^Negative.

^c^Nuclear expression pattern.

^d^Cytoplasmic expression pattern.

SOX2 staining was found either cytoplasmic or nuclear in a proportion of tumors while the majority showed both the localizations (Fig. 1). This prompted us to analyze SOX2 staining patterns separately. Apparent nuclear SOX2 staining was detected in 119 (71%) tumors and cytoplasmic staining in 141 (84%) tumors (Table 2). In normal urothelium there was generally a strong cytoplasmic staining throughout the urothelium and some positively stained nuclei mostly concentrated at the basal or suprabasal layer of urothelium (Fig. 1).

Ganglioside SSEA-4 was generally absent in normal urothelium while many tumor cells showed positive staining in the cytoplasm or partially at the plasma membrane (Fig. 1). For SSEA-4, H-score was greater than 0 in 64 (38%) tumors and 4 or more in 33 (19%) tumors (Table 2).

### Association with clinicopathological characteristics and cancer-specific survival

**Figure 2:**
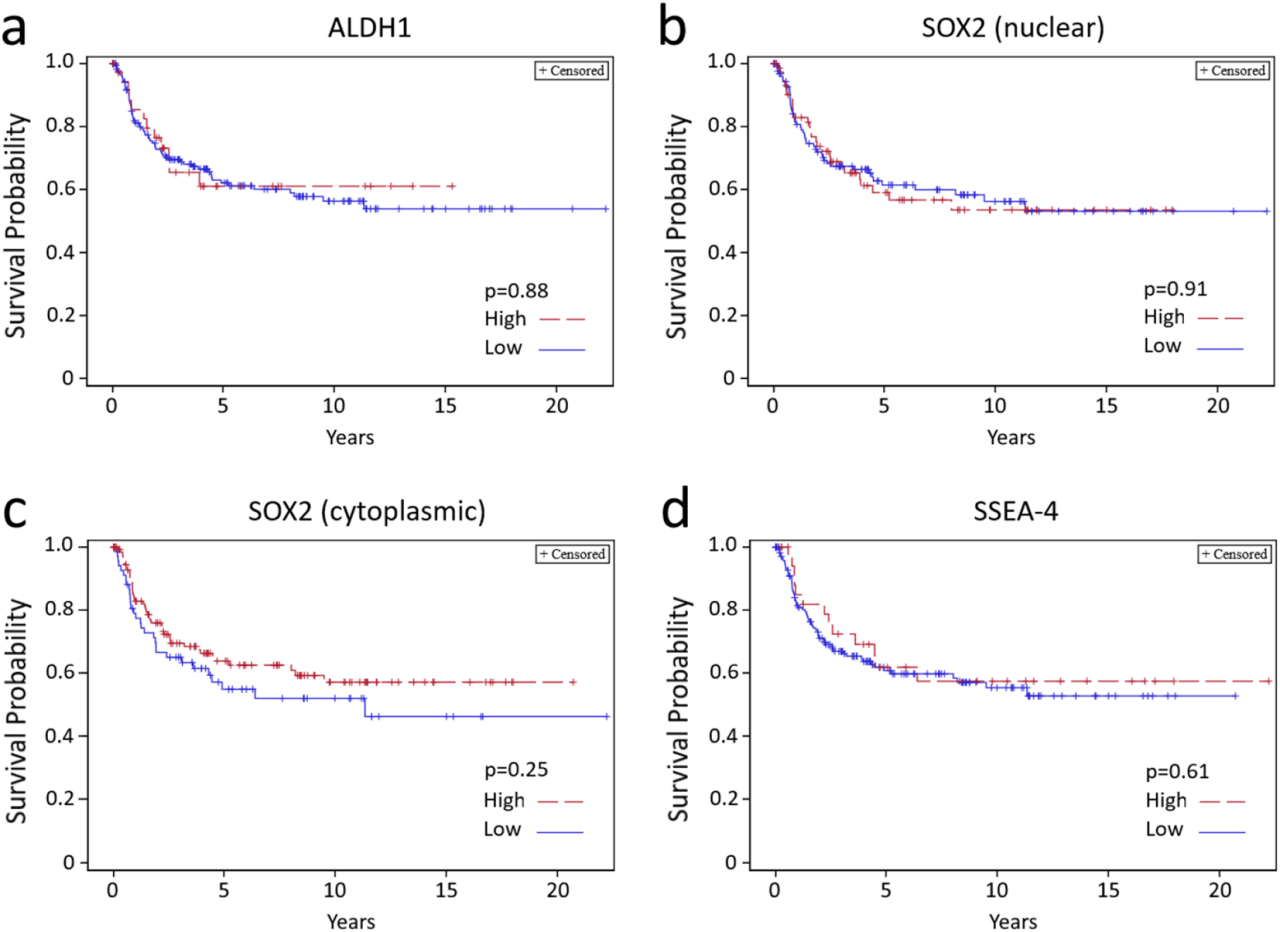
Kaplan–Meier survival analysis based on high (H-score ≥ 4) and low (H-score < 4) expression of ALDH1 (**a**), nuclear SOX2 (**b**), cytoplasmic SOX2 (**c**) and SSEA-4 (**d**) after RC in the RC-only cohort.

Based on previous published studies we first tested the prognostic value of ALDH1 in a RC cohort. H-score of 4 or more was not associated with carcinoma grade, stage (organ confinement) or lymphovascular invasion (LVI). Neither was there any significant difference in cancer-specific survival between the high and low expression groups (Table 3 and Fig. 2a).

**Table 3:** Staining associations with clinicopathological characteristics in the RC-only cohort.

Variable	LVI^a^		G3 vs. other (1973)^b^		Organ confined^c^	
	RR	95% CI	RR	95% CI	RR	95% CI
**ALDH1**						
Low (151)	Reference		Reference		Reference	
High (22)	0.98	0.55–1.78	1.25	0.94–1.68	0.92	0.64–1.33
**SOX2 (N)**						
Low (108)	Reference		Reference		Reference	
High (59)	0.92	0.61–1.40	1.04	0.82–1.33	1.12	0.88–1.42
**SOX2 (C)**						
Low (53)	Reference		Reference		Reference	
High (114)	0.64	0.43–0.93	1.09	0.84–1.42	1.38	1.02–1.86
**SSEA-4**						
Low (136)	Reference		Reference		Reference	
High (33)	1.01	0.62–1.67	0.53	0.33–0.85	1.27	1.00–1.61

Number of patients is shown in brackets for each subgroup. Comparison between high (H-score ≥ 4) and low (H-score < 4) expression. 95% Confidence intervals for risk ratio not including 1.0 are underlined.

^a^Lymphovascular invasion.

^b^Grade 3/3 according to WHO 1973 criteria.

^c^Organ confinement defined by pT class 2 or lower and absence of lymph node metastasis.

For cytoplasmic SOX2, a low H-score (3 or lower) was associated with LVI and non-organ confined disease but not with histological grade (Table 3). In support, high cytoplasmic SOX2 expression was associated with improved cancer-specific survival although this failed to show statistically significant difference (Fig. 2c). Nuclear staining of SOX2 showed no association with any clinicopathological characteristic or survival (Table 3 and Fig. 2b).

For SSEA-4, H-score of 4 or higher was inversely associated with high grade, but not with LVI or cancer-specific survival (Table 3 and Fig. 2d). In addition, H-score of 4 or higher was marginally associated with organ-confined disease (Table 3).

### Co-expression of ALDH1, SOX2 and SSEA-4 in bladder cancer

Potential co-expression of all the studied markers was further tested using data from all RC samples. 95% confidence interval for relative risk for high H-score (4 or more) was calculated between all markers. High ALDH1 staining intensity was marginally associated with nuclear SOX2. Furthermore, the nuclear SOX2 was associated with cytoplasmic SOX2 as expected. 95% Confidence intervals of risk ratios are shown in Table 4.

**Table 4:** Association of different markers in the RC-only cohort.

*95*% CI	ALDH1	SSEA-4	SOX2 (N)	SOX2 (C)
ALDH1	N/A	0.22–1.58	1.16–2.41	0.88–1.40
SSEA-4	0.22–1.58	N/A	0.42–1.28	0.82–1.34
SOX2 (N)	1.17–3.81	0.35–1.33	N/A	1.37–1.96
SOX2 (C)	0.68–2.57	0.59–2.15	2.05–7.9	N/A

Table shows 95% confidence interval of relative risk for high expression (H-score ≥ 4) of a marker on the column when expression of another marker on a row is high. For example, high expression of ALDH1 is associated with high nuclear expression of SOX2. Confidence intervals not including 1.0 are underlined.

### CSC markers as predictors of neoadjuvant chemotherapy

**Figure 3:**
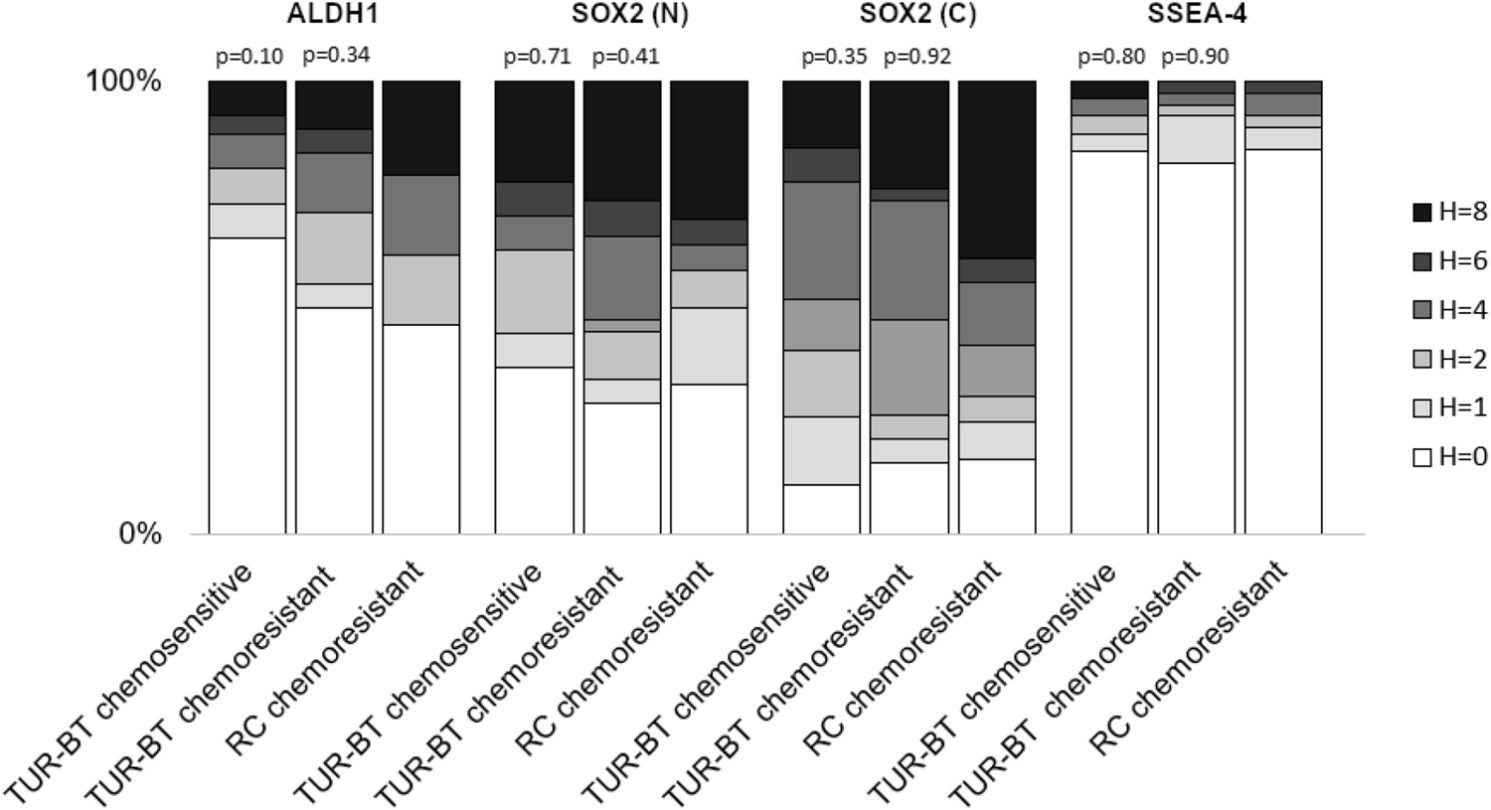
Distributions of H-scores before (TUR-BT) and after (RC) neoadjuvant chemotherapy. The first p-value denotes the statistical difference between TUR-BT chemosensitive and RC chemoresistant tumors while the latter denotes the statistical difference between TUR-BT chemoresistant and RC chemoresistant tumors.

TMA samples from TUR-BT operations prior NAC were available from 65 patients for ALDH1 and 66 patients for SOX2 and SSEA-4 staining. Of these patients, 39, 36 and 40 respectively had also residual tumor in RC specimens after NAC. For any of the markers tested, H-score 4 or higher did not predict response to NAC (Table 5). To test the hypothesis that cancer stem cells are enriched in chemoresistant tumors and further after chemotherapy due to drug resistance, we compared the distributions of different H-scores of both chemosensitive and chemoresistant primary tumors to chemoresistant RC residual tumors. There was an enrichment of high H-score tumors for ALHD1 and SOX2 (N and C) after NAC but these differences failed to show statistically significant difference (Fig. 3).

**Table 5:** Association between high expression (H-score ≥ 4) of markers in TUR-BT samples and neoadjuvant chemotherapy response, where low expression (H-score < 4) is reference.

Variable	Complete vs. other		Complete/Partial vs. other		Progression vs. other	
	RR	95% CI	RR	95% CI	RR	95% CI
**ALDH1**						
Low (48)	Reference		Reference		Reference	
High (17)	0.7	0.32–1.53	0.77	0.45–1.30	1.39	0.57–3.30
**SOX2 (N)**						
Low (36)	Reference		Reference		Reference	
High (30)	0.67	0.37–1.22	0.94	0.64–1.38	0.6	0.23–1.56
**SOX2 (S)**						
Low (33)	Reference		Reference		Reference	
High (33)	0.94	0.54–1.65	1.09	0.75–1.59	0.82	0.34–2.01
**SSEA-4**						
Low (62)	Reference		Reference		Reference	
High (4)	1.24	0.44–3.46	0.82	0.30–2.22	N/A^a^	N/A^a^

Number of patients is shown in brackets for each subgroup.

^a^There was no disease progression among the patients with high SSEA-4 H-score, and therefore the association with progression could not be tested.

## Discussion

In the present study, we evaluated the expression and prognostic and predictive value of stem cell markers ALDH1, SOX2 and SSEA-4 in two different MIBC cohorts treated either with RC only, or with RC after NAC. Based on previously published reports^14,17–19,28^ we originally hypothesized that BC patients whose carcinomas are enriched in CSCs would show less favorable clinicopathological characteristics, more frequent resistance to chemotherapy and shortened disease specific survival. Furthermore, if CSCs were in fact more resilient to chemotherapy, they were expected to enrich following NAC treatment. To our surprise, we found no strong evidence to support our initial hypotheses or previously published studies. The only exception was low cytoplasmic SOX2 staining, which was associated with LVI, non-organ confined disease and to some extent, shortened disease specific survival. To further confirm our findings, we repeated all the analyses using either staining intensity or proportion of positively stained cells alone as a read-out but found no major difference in the results (data not shown).

The association of ALDH1 with high clinicopathological grade, increased tumor size and poor differentiation has been found in multiple studies^17–19,29^. The same studies found an association between high ALDH1 expression and poor disease specific survival. For SOX2, a single study found an association between high expression and poor clinicopathological characteristics and survival in NMIBC^14^. Even though our results regarding the prognostic value of studied biomarkers were different from others’, the frequencies of both ALDH1 and SOX2 expression were congruent with previous studies. The reason for the different results of current and previous studies can only be speculated. However, it is important to note that SOX2 was previously evaluated in NMIBC cohort while ours consists of MIBC patients. Since NMIBCs more frequently harbor *FGF3* mutations and MIBCs are enriched in *TP53* and *Rb* mutations^30^ it is possible that these differences in molecular pathogenesis override the effects of CSC phenotype. While the patients’ mean age and smoking history in all the cohorts were relatively similar, it is also possible that differences in the expression of CSC markers and outcome are influenced by other risk factors and/or ethnic background of the patients. Equally, the differences in staining methods and antibody clones used may also explain the discordant results.

In many cancer types, such as oral squamous cell carcinoma and colorectal carcinoma both nuclear and cytoplasmic localizations of SOX2 have been previously reported while in lung squamous cell carcinoma and nasopharyngeal carcinoma nuclear localization appears to predominate^31,32^. The nuclear import and export of SOX2 is regulated by phosphorylation at Thr118^33^ and acetylation at Lys75^34^, respectively, and the phosphorylation of SOX2 is considered crucial for stem cell self-renewal or reprogramming^33^. Nevertheless, the data from different malignancies suggests that cytoplasmic and nuclear SOX2 may have diverse functions. Likewise, in our patient material both cytoplasmic and nuclear localizations were observed and associated with each other. Interestingly, low cytoplasmic staining was associated with LVI and non-organ confined disease, and to some extent, predicted less favorable survival after RC (Table 3 and Fig. 2C). Correspondingly, one would assume that high nuclear SOX2 staining indicates high SOX2 transcriptional activity and poor survival but this proved not to be the case and the more detailed action of SOX2 in different subcellular localizations of CSCs clearly warrants further studies.

To our knowledge, there is no previously published data about co-expression of ALDH1, SOX2 and SSEA-4 in BC, nor studies on enrichment of these markers post chemotherapy. Furthermore, ours is the first to investigate the role of SSEA-4 in BC patient material. Although all three are implicated in pluripotency of stem cells, they have vastly different cellular functions and signaling pathways^8–10,15,16,20^. Despite all of them being regarded as CSC markers and thus expected to reflect similar features in cancer cells, they were not significantly co-expressed in our study material. Further studies are needed to confirm this finding, but it appears clear that none of the CSC markers are universal and there may be multiple CSC subclones with different immunophenotypes within the tumors. Great individual variability in CSC number within tumors is commonly known and was predictably demonstrated in our results as well. Furthermore, there is still relatively little knowledge about the interactions of different CSC markers and the cellular mechanisms of their effects. CSCs are interacting with their surrounding non-malignant cells, and it can be speculated that some CSC biomarkers may display relevance to disease progression only within certain microenvironment. Taken together, a more detailed understanding of the underlying mechanisms of action of different CSC biomarkers could pave way for better biomarkers or sets of biomarkers in the future^4–6^.

Despite the pessimistic overall results, the strengths of this study include analysis of multiple CSC markers in parallel, a long follow-up time, low drop-out rate, and the inclusion of both neoadjuvant treated and chemotherapy naïve patients. The main weaknesses of our study are its retrospective nature, and the small sample size of NAC cohort. One could also question whether TMA material is representative regarding the overall expression of studied CSC markers within the tumor. However, we want to emphasize that up to three cores from each tumor were analyzed with highly similar results suggesting that the expression of studied markers is relatively uniform.

In conclusion, our results cast doubt on the reliability of ALDH1 and SOX2 as clinically relevant prognostic biomarkers in bladder cancer. We also conclude that SSEA-4 was neither prognostic nor predictive biomarker in our material, but this needs to be confirmed in the follow-up studies.

## Materials and methods

### Study population

The study population consisted of 195 RC patients treated at Turku University Hospital between 1985 and 2005 (RC cohort) and 74 patients who underwent TUR-BT prior to NAC and RC between 2007 and 2013 at either Turku University Hospital or Helsinki University Hospital (NAC cohort). All the patients included in the study had histologically confirmed MIBC (pT2 or higher) at TUR-BT and/or muscular invasion in imaging. The treatment was based on the European Association of Urology (EAU) guidelines at any given time. The clinical follow-up data was collected from hospital registries and survival data from the Finnish Cancer Registry. Informed consent was obtained from all subjects involved in the study.

### Tissue microarrays

Diagnostic formalin-fixed and paraffin-embedded tissue blocks were collected from pathology archives of the Turku and Helsinki University Hospitals. The tumors were reviewed by two expert uro-pathologists and classified based on both the WHO 1973 and WHO/ISUP 2004 classifications. Three tissue cores of 1 mm in diameter were punched from representative tumor areas for each patient and transferred into recipient tissue microarray (TMA) blocks. In addition, one tissue core from morphologically benign urothelium for each patient was included into TMA whenever available in original tissue blocks. TMA was created from RC and TUR-BT specimens in both cohorts.

### Immunohistochemistry

TMA sections were cut at four micrometer thickness, deparaffinized with xylene and rehydrated in graded series of alcohol. The sections were pretreated with Target Retrieval Solution (Dako) pH 6 (for SSEA-4) or pH 9 (for ALDH1 and SOX2), microwaved twice for 7 min and then washed three times with Tris–HCl buffer solution. Slides were next incubated in 3% hydrogen peroxide solution for 10 min, washed three times with Tris–HCl buffer solution and incubated for 10 min in Normal antibody diluent (Immunologic BD09-125). Mouse monoclonal IgG3 anti-human SSEA-4 antibody (clone MC-813-70, 1:1000, STEMCELL Technologies, Vancouver, Canada), mouse monoclonal IgG1 antibody against human ALDH1 aa. 7–128 (clone 44/ALDH, 1:200, BD Transduction Laboratories, NJ, USA) and mouse monoclonal IgG1 anti-human SOX2 (clone E-4, 1:500, Santa Cruz Biotechnology, Dallas, USA) were diluted in Normal antibody diluent and applied on the sections for 1 h. After washing three times with Tris–HCl buffer solution, the primary antibodies were detected with Brightvision + goat anti-mouse/rabbit HRP secondary antibodies (DPVB110HRP, Immunologic) for SSEA-4 and ALDH1A1, and EnVision + Dual Link HRP (Dako) for SOX2 for 20–30 min, followed by DAB substrate (Dako K3468) for 10 min. After washing three times with distilled water, the sections were counterstained with Mayer’s Hematoxylin Solution. BenchMark XT automated IHC/ISH slide staining system (Ventana Medical Systems, Inc.) was used for all the stainings and multiple staining conditions were tested for each antibody to achieve optimal staining results before staining the TMA material. Liver and testis were used as positive controls for ALDH1, benign urothelium for SOX2, and testis for SSEA-4. Lymphoid tissue was used as a negative control for all the antibodies.

All the slides were digitized using Pannoramic 250 scanner (3DHistech Ltd, Budapest, Hungary), viewed with CaseCenter software (3DHistech), and scored visually on a computer screen as follows: negative = 0, weak = 1, strong = 2. The most strongly stained carcinoma cells were first selected as a reference for high expression for each biomarker used. Carcinoma cells with no staining or equal to non-specific background were considered negative. Up to three carcinoma samples from each patient were analyzed and the cases with unsatisfactory tumor samples (e.g. tissue lost on the slide or no carcinoma cells in the TMA sample) were excluded from the final data analysis. The proportion of positively stained carcinoma cells was graded by approximation as follows: 0 for < 1% positive cells, 1 for 1–25% positive cells, 2 for 26–50% positive cells, 3 for 51–75% positive cells and 4 for 76–100% positive cells. Histological staining score (H-score) was calculated by multiplying the staining intensity score by the proportion score resulting in a number 0–8. In the final analysis, H-score of 4 or higher was considered high expression for each biomarker studied. Since morphologically benign tissue was not available from all the patients and these tissues may carry precancerous genomic alterations, the marker status in morphologically benign urothelium was not analyzed quantitatively.

### Statistical analyses

The Kaplan–Meier method was used in survival analysis in Fig. 2. Pearson’s chi-squared test was used in Fig. 3 and Tables 3, 4 and 5. In the NAC cohort (Fig. 3), the cases lacking carcinoma tissue in cystectomy specimen were considered chemosensitive whereas the cases with viable residual/recidive carcinoma in cystectomy specimen were considered chemoresistant.

### Institutional review board statement

The study was conducted according to the guidelines of the Declaration of Helsinki, and approved by the Research Ethics Board of the Hospital District of Southwest Finland (1.8.2006/301).

## Data Availability

The datasets generated during and/or analysed during the current study are available from the corresponding author on reasonable request.
